# Metabolic Effect of Estrogen Receptor Agonists on Breast Cancer Cells in the Presence or Absence of Carbonic Anhydrase Inhibitors

**DOI:** 10.3390/metabo6020016

**Published:** 2016-05-26

**Authors:** Anissa Belkaid, Miroslava Čuperlović-Culf, Mohamed Touaibia, Rodney J. Ouellette, Marc E. Surette

**Affiliations:** 1 Department of Chemistry and Biochemistry, Université de Moncton, Moncton, NB E1A 3E9, Canada; Anissa.Belkaid@vitalitenb.ca (A.B.); mohamed.touaibia@umoncton.ca (M.T.); marc.surette@umoncton.ca (M.E.S.); 2 Atlantic Cancer Research Institute, Moncton, NB E1C 8X3, Canada; RodneyO@rrsb.nb.ca; 3 National Research Council of Canada, 1200 Montreal Road, Ottawa, ON, Canada

**Keywords:** 17β-estradiol, ferulic acid, carbonic anhydrase, ER + cells, metabolomics, cancer metabolism, acetazolamide

## Abstract

Metabolic shift is one of the major hallmarks of cancer development. Estrogen receptor (ER) activity has a profound effect on breast cancer cell growth through a number of metabolic changes driven by its effect on transcription of several enzymes, including carbonic anhydrases, Stearoyl-CoA desaturase-1, and oncogenes including HER2. Thus, estrogen receptor activators can be expected to lead to the modulation of cell metabolism in estrogen receptor positive cells. In this work we have investigated the effect of 17β-estradiol, an ER activator, and ferulic acid, a carbonic anhydrase inhibitor, as well as ER activator, in the absence and in the presence of the carbonic anhydrase inhibitor acetazolamide on the metabolism of MCF7 cells and MCF7 cells, stably transfected to express HER2 (MCF7HER2). Metabolic profiles were studied using 1D and 2D metabolomic Nuclear Magnetic Resonance (NMR) experiments, combined with the identification and quantification of metabolites, and the annotation of the results in the context of biochemical pathways. Overall changes in hydrophilic metabolites were largest following treatment of MCF7 and MC7HER2 cells with 17β-estradiol. However, the carbonic anhydrase inhibitor acetazolamide had the largest effect on the profile of lipophilic metabolites.

## 1. Introduction

Cancers, including breast cancer, remain a major challenge for healthcare. The complexity and adaptability of this disease continues to evade the development of effective and safe therapies. In the era of personalized medicine, the high throughput analysis of cancer metabolism under different challenges, *i.e.* metabolomics, is expected to provide significant novel information and tools for the analysis of drug resistance, which remains one of the major clinical setbacks in cancer treatment [1,2]. Breast cancer is a heterogeneous disease with different subtypes presenting distinct cellular and molecular characteristics. The presence or absence of a number of hormone receptors in breast cancer subtypes is an important indicator used for the optimization of therapeutic strategies [3]. Hormone receptors defining breast cancer subtypes are estrogen receptor alpha (ERα), progesterone receptor (PR) and the human epidermal growth factor receptor 2 HER2/neu (HER2 or ERBB2). These receptors may be present individually or in various combinations, which may provide information into the aggressiveness of the tumor and determine the therapeutic strategy [4,5,6,7]. ERα plays a crucial role in the development of hormone-dependent breast cancer and is present in more than 70% of breast tumors.

ERα, once activated with estradiol or other agonists, acts both directly as a transcription factor and indirectly by the modulation of other pathways involved in chromosome replication, cell cycle regulation, cell survival, and growth factor signaling [8,9]. The activation of the ERα pathway by estradiol increases cell proliferation and induces many genes directly involved in metabolism, such as glycolytic and lipogenic enzymes. Similarly, HER2 expression is also associated with enhanced lipogenesis. The transcription factor activity of ERα regulates the expression of metabolic enzymes that are providers of building blocks for cellular growth [10,11]. One of these ERα targets is stearoyl-CoA desaturase-1 (SCD1) [12]. SCD1 is the principal supplier of monounsaturated fatty acids that are necessary for optimal membrane fluidity and membrane biogenesis and has emerged as a potential therapeutic target for lung, prostate, and breast cancer [12,13,14]. Estradiol activation of ERα also leads to increased expression of carbonic anhydrase XII [15,16,17]. Carbonic anhydrases (CA) are a family of 10 isoenzymes with different enzymatic properties and various subcellular localizations [18]. CA are metaloenzymes that form bicarbonate from a reversible hydration of CO_2_, thereby regulating the microenvironment acidity and tumor malignant phenotype [19]. In addition, CA modulates tumor microenvironment acidity by supporting lactate flux in cancer cells [20], thus the inhibition of CA isozymes is a promising anti-cancer therapy [20,21].

Ferulic acid (FA, 4-hydroxy-3-methoxy cinnamic acid) is an active compound derived from *Angelica sinensis*, known to have several biological activities, including antioxidant, anti-inflammatory, anti-cancer, and anti-apoptotic properties [22]. Moreover, FA can activate ERα in a manner that has been shown to be comparable to that of estradiol, stimulating MCF7 mammary carcinoma cell proliferation and inducing increased expression of both HER2 and ERα genes [23]. Interestingly, FA also has significant antioxidant effects, and can inhibit several metalo-enzymes, including CA [16,17].

In this study, the impact of estradiol and FA, both ERα activators, on breast cancer cell metabolism was investigated with and without CA inhibition using the pan-CA inhibitor acetozolamide. The effects on metabolism were measured in the ER positive MCF7 breast carcinoma cell line using a previously-developed NMR-based metabolomics method [24,25,26] for both quantitative and qualitative analyses of hydrophilic and lipophilic cellular extracts.

## 2. Results and Discussion

**Figure 1:**
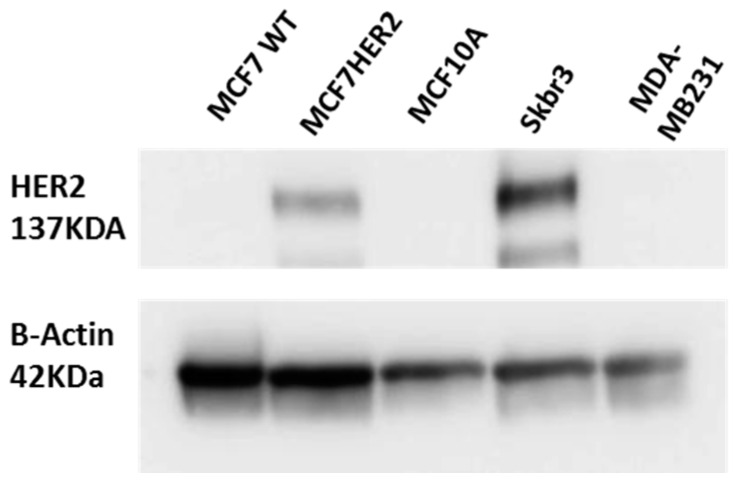
Validation of HER2 status in HER2 stably transfected MCF7 cells (termed MCF7HER2). Immunoblot analysis of HER2 expression in MCF7, MCF7HER2 and MCF10A cells. SKBR3 cells are used as a positive control and MDA-MB231 cell line as a negative control for HER2 expression. Presented immunoblots show a representative of 3 independent experiments.

**Scheme 1:**
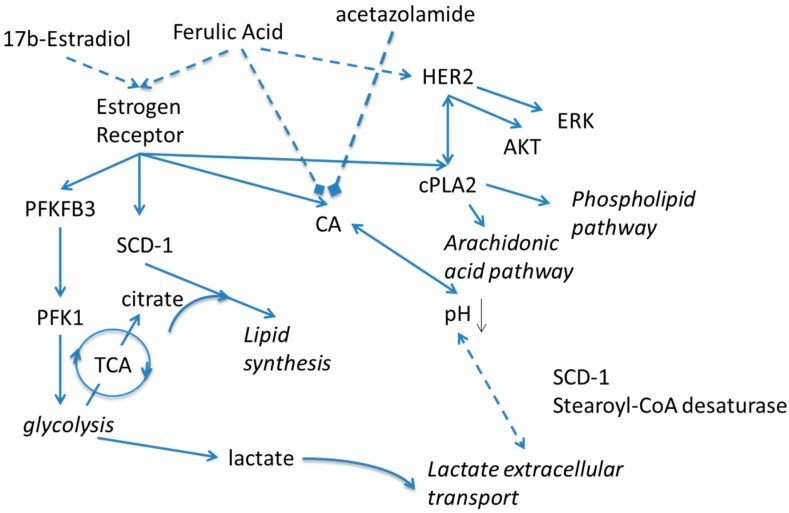
Schematic presentation of proposed effects of Estradiol and carbonic anhydrase on metabolism. Solid lines indicate known relationships through metabolic pathways or protein-protein interactions. Dashed lines indicate activating or inhibiting chemical actions. PFKFB3—6-Phosphofructo-2-Kinase/Fructose-2,6-Biphosphatase 3; PFK1—Phosphofructokinase-1; TCA—Trichloroacetic acid cycle, Krebs cycle; SCD-1—Stearoyl-CoA desaturase-1; CA—carbonic anhydrase; HER2—human epidermal growth factor receptor 2; ERK—Extracellular Signal-regulated Kinase; AKT—Protein kinase B; cPLA2—Cytosolic phospholipases A2.

The present study investigates metabolic differences between the ERα +/low HER2 mammary carcinoma cell line MCF7 and MCF7 cells stably transfected with HER2 (MCF7HER2) (ERα +/high HER2) (Figure 1), with the immortalized MCF10A normal mammary epithelial line used as an estrogen insensitive control [12]. Metabolism in these cells was compared in untreated controls and following incubation with 17β-ED or FA, in the presence or absence of the CA inhibitor acetazolamide. The effect of these compounds on the three cell lines was investigated through the analysis of changes in cellular metabolic profiles using NMR ^1^H analysis with methods previously developed and used by our group [24,25,26]. Previously indicated relations between the tested molecules and some of the major proteins responsible for cancer progression and metabolism are schematically presented (Scheme 1) and were explored through metabolomics analysis in this work.

**Figure 2:**
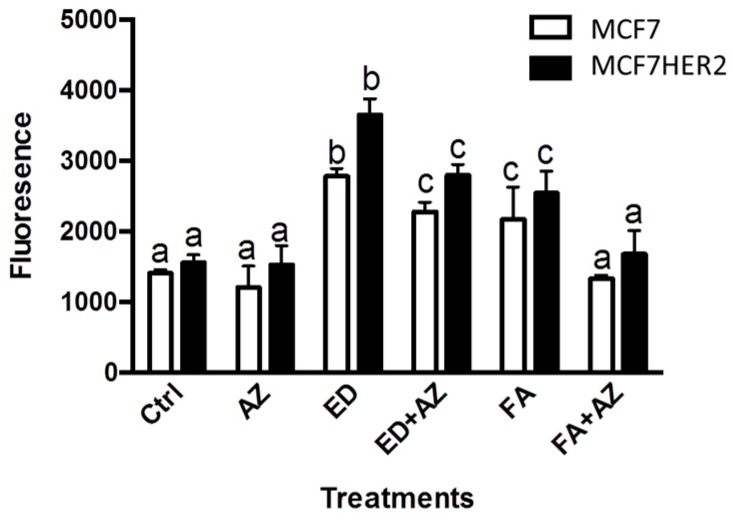
17β-estradiol induces cell proliferation in both MCF7 and MCF7HER2. Cells were incubated with 2 nM 17β-estradiol (ED), 10 μM ferulic acid (FA) and 100 μM acetazolamide (AZ) and cell proliferation was measured as described in the Experimental Section. Boxplots show means ± SEM of 6 independent experiments. Values for the same cell line with different superscripts are significantly different (*p* < 0.05) as determined by one way ANOVA with subsequent Tukey’s *post hoc* test.

17β-estradiol (ED) is an estrogen receptor activator that induces cell division in ER+ breast cancer cells. This effect is clearly confirmed in both MCF7 and MCF7HER2 cell lines where incubation with ED causes significant increases in cell proliferation in both MCF7 and MCF7HER2 cells (Figure 2). Ferulic acid treatment also increased cell proliferation in both MCF7 and MCF7HER2 cells (Figure 2), in agreement with previous studies [23,27]. Addition of the pan-CA inhibitor acetazolamide leads to a slight decrease in cell proliferation in both cell lines, in agreement with the previously observed anti-proliferative effect of CA inhibitors or CA knockdown [20,28]. In MCF10A cells, cellular proliferation was not affected by the treatments with ED, FA or acetazolamide (data not shown). The presence of CA in MCF7 cells has been previously established [15,20,29] and although the role of CA is particularly pronounced in hypoxia [20], CAs are expressed in cancer cell lines, even under normoxic conditions, where metabolism is enhanced due to the Warburg effect, with roles in pH regulation [30] and lactate transport to the extracellular medium [20]. Indeed removal of lactate from metabolically active cells is required to prevent intracellular acidification and a reduction in cell metabolism and proliferation [20].

**Figure 3:**
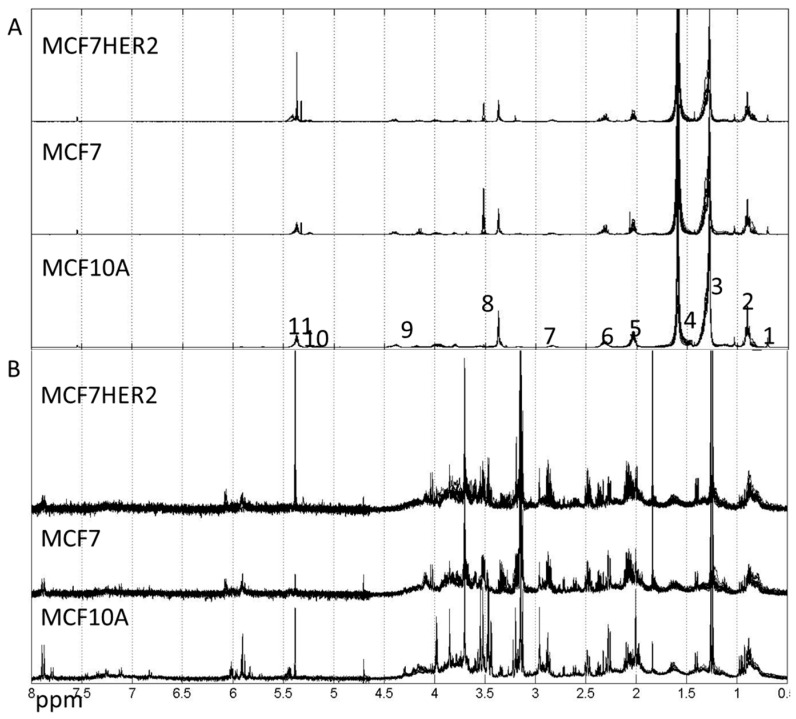
Spectra of lipophilic (**A**) and hydrophilic (**B**) metabolic extracts from MCF10A, MCF7 and MCF7HER2 cells incubated under different treatment conditions in different biological replicates measured here. (A) Labels correspond to peaks from: 1–CH3; 2–CH3 (L, Ch), CH2 (Ch); 3–CH2 (L, Ch); 4–CH2CH2COO (L) and H2O; 5–CH2CH=CH (L); 6–N(CH3); 7–Ar-H; 8–N(CH3)3; 9–CH2OCOR, CH2OPO2; 10–CHOCOR (lipids); 11–HC=CH (lipids, cholesterol). Assignment of peaks is based on [31]. (B) Hydrophilic spectra for the same three cell types. These spectra are representative of 3–5 independent experiments.

Metabolic profiles for hydrophilic and lipophilic extracts were measured for all three cell lines (MCF10A, MCF7 and MCF7HER2) under studied treatments, see spectra in Figure 3.

**Figure 4:**
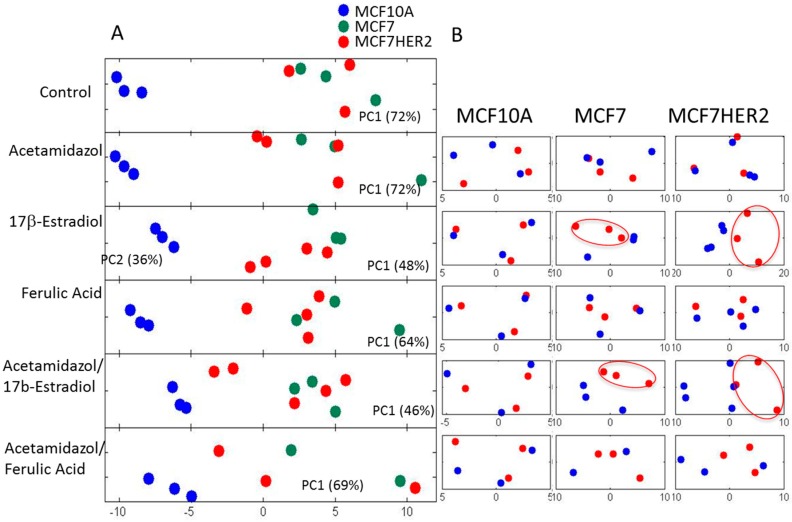
PCA of spectra of hydrophilic profiles for samples in three cell lines with different treatments. (**A**) PCA of spectra of hydrophilic profiles. (**B**) Individual comparisons between control (blue dots) and treated samples (red Dots) in the three cell lines. PC1—principal component 1 score shows percentage of variance between samples that is represented by PC1 axes; PC2—principal component 2 shows percentage of variance between samples that is represented by PC2 axes. Percentage separation was shown wherever there is a visible separation between sample groups. Separation of sample groups in any principal component axis is more significant if the percent score is larger. The third replicate in the acetamidazol/ferulic acid treatment of MCF7 cells was not included due to the quality of the NMR signal.

Overall metabolic profile changes are best observed from the analysis of spectra. Principal component analysis (PCA) of the measured hydrophilic metabolites for the three cell lines incubated under different treatment conditions is shown in Figure 4A. In the untreated control condition, MCF10A cells had a highly distinct profile from that of the other cell lines, with no significant difference in the major variances between MCF7 and MCF7HER2 cells. A similar overall result was observed following incubations with acetazolamide and ferulic acid. Treatment with 17β-estradiol created change in the metabolic profile between all three cell types with significantly reduced difference between MCF10A and MCF7 and MCF7HER2 cells. According to PCA shown in Figure 4A, major PC1 variance between these three groups goes from over 70% in control group to under 50% in 17β-estradiol treated cells. At the same time PC2 component shows variance between MCF7 and MCF7HER2 cells only in 17β-estradiol treated cells. Analysis of treatments and controls for each cell line (Figure 4B) show no significant effect on the overall profile in MCF10A cells for any of the tested treatments. In MCF7 and MCF7HER2 cells, 17β-estradiol once again had the most profound effect on PCA spectra when applied alone or in combination with acetazolamide resulting in significant changes from untreated controls. There was no measured effect on PCA of metabolic profiles following treatment of cells with ferulic acid or acetazolamide.

**Figure 5:**
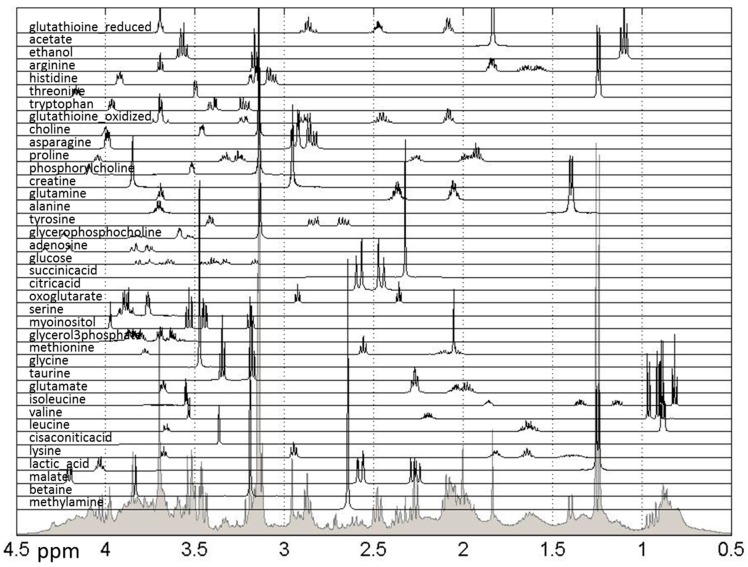
Reference spectra of metabolites used for quantification of relative concentrations of hydrophilic metabolites measured in NMR spectra. Shown are all quantified metabolites as well as the sum of NMR spectra for all samples. The spectral range from 0.5 to 4.5 ppm is shown, however the spectral range used for quantification was 0.5–9 ppm.

Further biological analysis of the effects of treatments can be performed using quantified metabolic data. Methods previously developed and utilized by our group [24,25,26,32] were used to quantify 38 metabolites previously listed as present in breast cancer cells using publically-available spectra, as shown in Figure 5.

**Figure 6:**
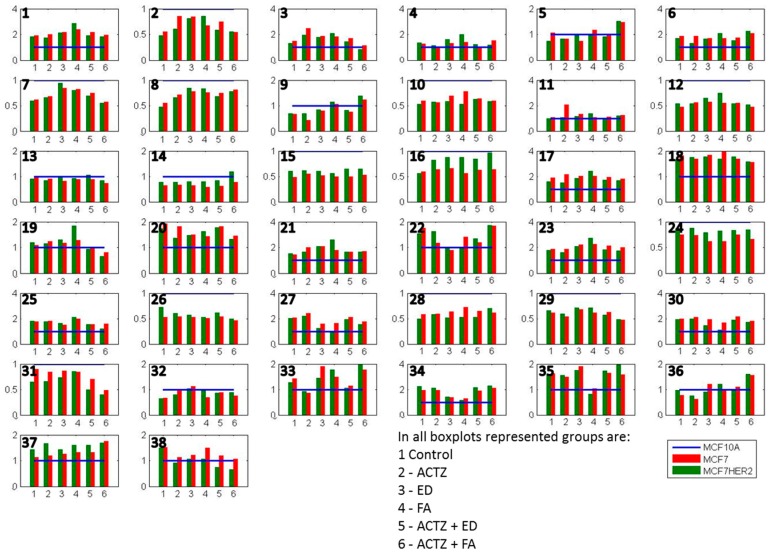
Average concentrations of metabolites in hydrophilic samples of three cell lines incubated in 6 different treatment conditions. All concentrations are shown relative to MCF10A concentrations that are represented by a normalized blue line with a value of 1. Shown metabolites are: 1 methylamine; 2 betaine; 3 malate; 4 lactic_acid; 5 lysine; 6 cisaconiticacid; 7 leucine; 8 valine; 9 isoleucine; 10 glutamate; 11 taurine; 12 glycine; 13 methionine; 14 glycerol-3-phosphate; 15 myoinositol; 16 serine; 17 oxoglutarate; 18 citricacid; 19 succinicacid; 20 glucose; 21 adenosine; 22 glycerophosphocholine; 23 tyrosine; 24 alanine; 25 glutamine; 26 creatine; 27 phosphocholine; 28 proline; 29 asparagine; 30 choline; 31 oxidized glutathione (GSSG); 32 tryptophan; 33 threonine; 34 histidine; 35 arginine; 36 ethanol; 37 acetate; 38 reduced glutathione (GSH). Values are the averages of 3 to 5 independent experiments.

This relative comparison of the effects of different treatments on metabolite concentrations in three cell lines allows us to focus on changes caused by specific differences between cell lines. Figure 6 and Supplementary Table S1 present the average concentrations of 38 metabolites in MCF7 and MCF7HER2 cells in the six treatment conditions relative to the concentrations in MCF10A cells undergoing the same treatment. Concentration differences are apparent for all metabolites in all conditions between malignant cell lines (MCF7 and MCF7HER2) and the immortalized normal MCF10A cell line. Relative metabolic differences between the immortalized normal MCF10A cell line and malignant cell lines (MCF7 and MCF7HER2) are shown in Supplementary Figure S1 for all metabolites, and major metabolic changes are determined using Statistical Analysis for Microarrays method (SAM) and are shown in Supplementary Figure S2.

Several metabolites have lower concentrations in both MCF7 and MCF7HER2 cells in all treatments compared to MCF10A cells. These include a number of amino acids including some branched chain amino acids (leucine, valine, glutamate, glycine, methionine, serine, alanine, creatine, proline, asparagine, tryptophan), as well as betaine, glycerol-3-phosphate (G3P), myoinositol, GSSG. At the same time methylamine, malate, lactic acid cis-aconic acid, citric acid, glucose, adenosine, glycerophosphocholine (GCP), tyrosine, glutamine, phosphocholine (PC), choline, histidine, arginine and ethanol are consistently more concentrated in cancer cell lines. In general the two breast cancer cell lines responded similarly to treatments with the most apparent difference between the effects of treatments on MCF7 relative to MCF7HER2 cells being in concentration changes of serine. In order to put the observed differences into the context of cellular metabolism, we show relative metabolite concentration changes following individual treatments in MCF7 and MCF7HER2 cells relative to the equivalent treatment effect in MCF10A cells.

**Figure 7:**
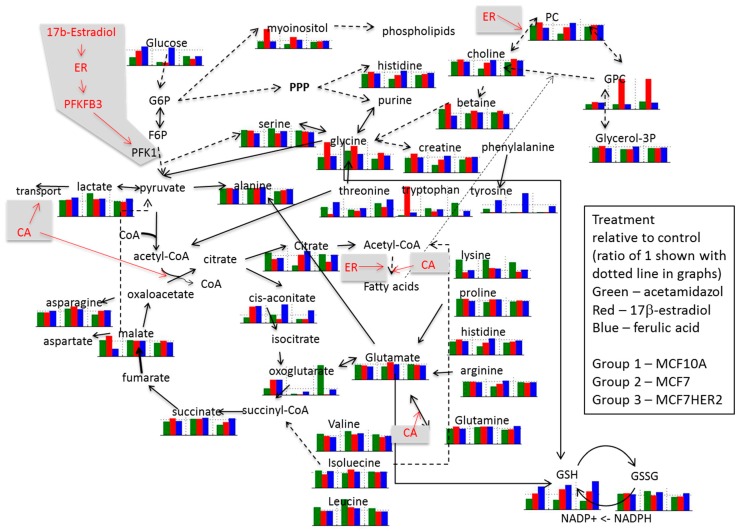
Schematic presentation of proposed effects of ER agonists and CA inhibition on metabolism. Imbedded graphs show metabolite concentration changes following acetazolamide, 17β-estradiol and ferulic acid treatment in MCF10A, MCF7 and MCF7HER2 relative to untreated controls in the same cell lines. Horizontal dotted line in each graph shows a value of 1 (relative effect value in untreated cells). Dashed arrowed lines show indirect (through several steps in the pathway) connection between metabolites; solid arrowed lines show direct pathway connection between metabolites. Values are from 3 to 5 independent experiments.

Relative concentrations for observed metabolites involved in glycolysis, Krebs cycle, and choline pathways as well as related amino acids are shown for different treatments in Figure 7. Imbedded graphs show metabolite concentration changes following acetazolamide, 17β-estradiol and ferulic acid treatment in MCF10A, MCF7, and MCF7HER2 relative to untreated controls in the same cell lines.

In the untreated control group (Figure 6) the glycolysis pathway including lactate production is enhanced in the cancer cell lines compared to MCF10A cells. In addition, concentrations of Krebs cycle intermediates are also increased in MCF7 cells possibly resulting from increased utilization of glutamate (with its concentration decrease) or from other amino acids (e.g., branched amino acids) also showing reduced concentrations compared to MCF10A cells. Increases in Krebs cycle further leads to enhanced citrate production as well as possibly contributing additionally to lactate production. Higher concentrations of histidine in the cancer cell lines possibly indicates increased PPP pathway as previously noted [33,34,35]. Average concentration change for lactic acid, succinic acid and histidine across different groups of samples are shown in in Supplementary Figure S3. Additionally, choline concentrations as well as concentrations of phosphocholine (PC) and glycerophosphocholine (GPC) are increased in cancer cell lines, once again in agreement with previous results.

Addition of 17β-estradiol leads to a number of concentration changes between these three cell lines with differences in behavior when compared against normal MCF10A cells, as well as when comparing MCF7 and MCF7HER2 cells. MCF7 cells respond to estradiol by increasing the consumption of glucose, possibly increasing the flux through the PPP cycle with a noted relative increase in histidine concentration. The effect of estradiol on Krebs cycle is more significant in MCF7HER2 cells with higher increases in the concentration of all Krebs cycle intermediates except citrate, possibly due to the ER activation of fatty acid synthesis. The effect of estrogen receptor activation also leads to significant increases in relative concentrations of choline as well as phosphocholines and glycerophosphocholines and a decrease in betaine in MCF7 cells but not MCF7HER2 cells. Production of lactate increases in both MCF7 and MCF7HER2 cells.

Treatment with ferulic acid leads to lesser change in relative concentrations, however, major significant additional changes are the same as in 17β-estradiol treated cells. Once again lactate production is increased particularly in MCF7HER2 cells. Serine concentration is also increased in MCF7HER2 cells. Choline and phosphocholine are not significantly affected, however there is an increase in the relative concentration of glycerophosphocholine in MCF7HER2 cells. HER2 activated MCF7 cells can represent a hybrid between MCF7 and SKBR3 cells and treatment with hypothetic estrogen receptor activators can lead to activation of PLA_2_ expression, altering ratio the between PC and GPC [36].

Treatment with the CA inhibitor acetazolamide does not alter relative metabolite concentrations between the three cell lines significantly for the majority of metabolites. Major changes are in lysine and citrate levels with an increase in concentration in MCF7 cells relative to the other two cell lines, suggesting an effect of CA inhibition on fatty acid biosynthesis in this cell line. Concentrations of lactic acid increased the most in MCF7HER2 cells (Supplementary Figure S3) possibly due to the reduced efflux out of the cell and this observation will need to be further investigated. Addition of acetazolamide to estradiol treatment leads to GPC concentration changes with a major decrease in MCF7 cells relative to cells treated only with estradiol as well as control cells. Choline and phosphocholine are directly related through the Kennedy pathway and their comparable relative concentrations are apparent in all treatment cases. Glycerophosphocholine can result from phosphatidylcholine in a PLA_2_-driven reaction further producing choline and glycerol-3-phosphate. Combined treatment with acetazolamide and ferulic acid leads to major concentration increases in lactate in MCF7 cells possibly indicating reduced efflux out of cells. As there is a reduction in the relative concentration of serine in MCF7 cells it can also be hypothesized that glycolysis in these cells increasingly goes to the lactic acid shunt, which is corroborated by decreased relative concentrations of several Krebs cycle metabolites. Concentrations of choline as well as PC and GPC show relative increases in MCF7 cells in this case while choline, PC and GPC concentration changes were observed in ER activated cells.

**Figure 8:**
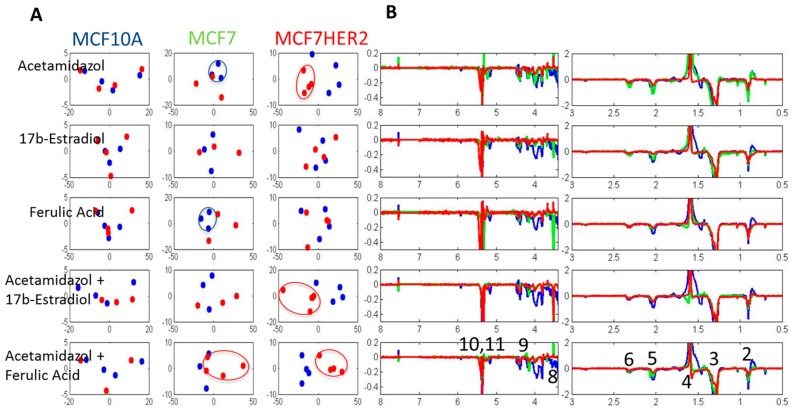
PCA of spectra of lipophilic metabolite for control (blue dots) and treated samples (red dots) in three cell lines. Labels correspond to peaks from: 1–CH3; 2–CH3 (L, Ch), CH2 (Ch); 3–CH2 (L, Ch); 4–CH2CH2COO (L) and H2O; 5–CH2CH=CH (L); 6–N(CH3); 7–Ar-H; 8–N(CH3)3; 9–CH2OCOR, CH2OPO2; 10–CHOCOR (lipids); 11–HC=CH (lipids, cholesterol). Assignment of peaks is based on Reference [31].

Changes in lipid profiles are apparent from the analysis of NMR metabolic profiles of lipophilic metabolites. Figure 8 shows PCA of spectra for the three cell lines comparing control and treated samples. The largest differences are in acetazolamide-treated cancer cells.

According to the trace analysis of PC1 (Figure 8B), the most significant variances leading to the observed separation of control and acetazolamide-treated MCF7HER2 cells are in CH2 peak of lipids or cholesterols, CH2CH2COO and CHOCOR peak of lipids, and HC=CH peak of lipids and cholesterols. The observed differences suggest increases in lipid concentrations and a decrease in cholesterol concentrations following acetazolamide treatment.

**Figure 9:**
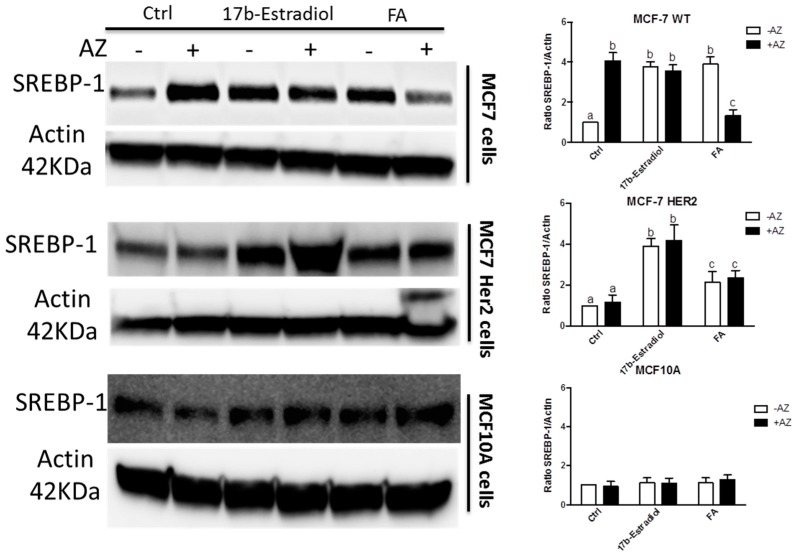
SREBP-1 expression profile in the 3 cell lines. Immunoblot analysis of SREBP-1 expression in MCF-7, T47D and MCF-10A cells incubated with 2 nM, 10 μM and 100 μM of 17β-estradiol, ferulic Acid (FA) and acetazolamide (AZ), respectively, or with vehicle EtOH and DMSO (Ctrl). The graphs show densitometry quantification of the SREBP-1 blots in the 3 cell lines. Immunoblots are representative of 3 independent experiments. Data are means ± SEM, n = 3 independent experiments. Values that have a different superscript are significantly different (*p* < 0.05) as determined by one-way ANOVA test with subsequent Tukey’s *post hoc* test.

To investigate this observation, we measured the expression of sterol-response element binding protein-1 (SREBP-1) as shown in Figure 9 under different conditions. SREBP-1 is a transcription factor that shows higher activity in the induction of genes involved in fatty acid synthesis than those participating in cholesterol synthesis [37,38]. As expected, the activation of ERα with both agonists, 17β-estradiol and FA, enhances the expression of SREBP-1 in both MCF7 and MCF7HER2 cell lines compared to untreated controls. The pan-CA inhibitor acetazolamide alone induces SREBP-1 expression in MCF7 cells but not in MCF7HER2 cells. Further lipidomics analysis is warranted in order to explore in more detail the effect of these treatments on lipid metabolism.

## 3. Experimental Section

### 3.1. Reagents

Cell culture media (DMEM/F12, RPMI-1640 and phenol red free-media) and charcoal stripped FBS were purchased from Hyclone Fisher. Normal FBS was from Wisent and penicillin/streptomycin were from Lonza. 17beta-estradiol (17β-ED), deuterium oxide and DMSO were purchased from Sigma-Aldrich. The ferulic acid and acetazolamide were from Biovision and Cedarlane Labs, respectively.

### 3.2. Cell Culture and Western Blot

MCF7 and MCF10A cell lines were purchased from the American Type Culture Collection (Manassas, VA). The MCF7HER2 cells were generously provided by Dr L.C. Murphy from the University of Manitoba. MCF7 and MCF7HER2 cells were cultured in RPMI 1640 supplemented with 10% FBS. MCF10A cells were cultured in DMED/F12 supplemented with 100 ng/mL cholera toxin, 10% FBS, 100 U/mL penicillin and 10 µg/mL streptomycin. The three cell lines were maintained at 37 °C in a humidified environment with 5% CO_2_. Before treatments cells were cultured for one week in phenol red-free medium supplemented with 10% charcoal-stripped FBS to starve cells from steroid hormones as previously described [12]. The cells were then treated for 5 days with the ER agonists FA (10 μM) or 17β-ED (2 nM). Acetazolamide (100 μM) was then added to the culture medium for one more day prior to analyses. 17β-ED was dissolved in ethanol, ferulic acid and acetazolamide were both prepared in DMSO.

### 3.3. Cell Viability and Western Blot

5 × 10^3^ cells were seeded in 96-well plates and cultured under the conditions described above. Cell viability was assessed using Cell titer blue (Promega) following the manufacturer’s instructions. Briefly, 20 μL of Cell titer blue was added to 100μl of media containing the treated cells and incubated for 1h at 37 °C. Then the microplate was subjected to analysis (544nm excitation/590nm emission) on a fluorescence microplate reader (VarioSkan, Thermo Scientific Corporation,). For Western blots, trypsinized MCF7, MCF7HER2 and MCF10A cells were washed in cold PBS and lysed in 50 mM tris-HCl pH 7.6, 150 nM NaCl, 2 mM EDTA and 1% Nonidet-P40 containing a cocktail of protease inhibitors (Roche). Proteins were quantified with the EZQ Protein Quantitation kit (Molecular Probes) and were separated on 4%–15% polyacrylamide Criterion TGX precast gel (Bio Rad). The separated proteins were transferred to polyvidine difluoride membranes and HER2 and SREBP-1 were detected using Cell Signaling (cat # 3250S) and BD Biosciences (Cat#557036) antibodies, respectively.

### 3.4. Hydrophilic and Lipophilic Metabolites Extraction

The hydrophilic metabolites were extracted as previously described [32]. Briefly, the treated cells were trypsinized, rinsed twice with PBS, centrifuged at 300× *g* for 1-min, and the pellet was kept on ice for 5 min. The pellets were resuspended in 1 mL 50% (v/v) acetonitrile/water, incubated on ice for 10 min, centrifuged at 16,000× *g* for 10 min at 4 °C, and the resulting supernatant was collected and evaporated under a stream of N_2_.

For the lipophilic metabolites the treated and trypsinized cells were washed in PBS and resuspended in 96 mL cold methanol/water (3.36/1, v/v) as previously described [25] . Samples were sonicated 3 times with 1-min cycles and 1-min wait periods. Cold chloroform was then added to the suspension in glass tubes and stored overnight at 4 °C. The following day cold chloroform and cold water (3.7 mL/pellet each) were added to the tubes, and samples were vortexed for 30 s. Homogenates were centrifuged at 200× *g* for 5 min at 4 °C. The lower phase containing the lipophilic metabolites was recuperated and dried down under a stream of N_2_ [39].

### 3.5. NMR Experimentation and Preprocessing

The dried hydrophilic and lipophilic residues were immediately dissolved in 0.7 mL of deuterium oxide or deuterated chloroform, respectively, and pipetted into a 5 mm NMR tube for NMR analysis. All ^1^H NMRs were performed on a Bruker Avance III 400 MHz spectrometer at 298 K. One-dimensional spectra for the hydrophilic fractions were obtained using a gradient water presaturation method with 512 scans, while the spectra of the lipophilic fractions were obtained using a regular proton experiment with 128 scans. Unless otherwise indicated, all analyses were performed on samples from 5 independent experiments performed on different days.

NMR spectra were processed using Mnova 9.1.0. Spectral preprocessing for hydrophilic spectra included exponential apodization (exp 1), global phase correction, and normalization using the total spectral area. Spectral regions from 0.5 to 9.5 ppm were included in the normalization and analysis. For lipophilic spectra preprocessing included exponential apodization (exp 1), global phase correction including manual correction when needed, baseline corrected using Whittaker Smoother, and normalization using the total spectral area. Spectral regions from 0.5 to 8 ppm (excluding 7.0–7.5ppm solvent peak) were included in the normalization and analysis.

Data pre-processing, including data organization, removal of undesired areas and binning, as well as data presentation were performed with Matlab (Matworks Inc.). Minor adjustments in peak positions (alignment) between different samples were performed using in-house alignment software (GASP), as well as Icoshift [40] The qualitative analyses of the major variances in the spectra were performed by using principal component analysis (PCA), as well as fuzzy k-means cluster analysis using the MATLAB platform. Feature selection was performed with the Significance Analysis for Microarrays (SAM) method [41].

### 3.6. Metabolite Quantification

Peak assignment was performed using several methods developed in our group and elsewhere and was based on metabolic NMR databases [42,43] Spectra for around 40 metabolites used in quantification were obtained from the Human Metabolomics Database [42] or the Biological Magnetic Resonance Databank [43] and were further analyzed visually and compared to the obtained spectra. An automated method for quantification based on multivariable linear regression of spectra with appropriately aligned metabolite data from databases was previously described in detail [24,25,26] and was used in this study. The assumption behind this approach is that the spectrum of a mixture is the same as the combination (sum) of spectra of individual components measured under the same conditions. Relative metabolite concentrations were estimated using nonlinear curve-fitting with the multivariate least-squares approach. The linear regression result was used as the starting point, and the model was constrained to concentrations: c ≥ 0. The NMR spectra of the mixtures (samples) are modeled as a sum of spectra for components (metabolites) in the mixture. Metabolite concentrations have been normalized across samples (assuming equal total metabolite concentration). For visualization, all samples were scaled across metabolites. All metabolite concentrations for all experiments are available from the authors.

## 4. Conclusions

NMR metabolomics analysis was used to investigate the effects of 17β-estradiol, an ER activator and ferulic acid, a CA inhibitor as well as ER activator, in the absence and in the presence of the CA inhibitor acetazolamide on the metabolism of MCF7 and MCF7HER2 cells. Overall changes in hydrophilic metabolites were largest following treatment of MCF7 and MC7HER2 cells with 17β-estradiol. However, the CA inhibitor acetazolamide has the largest effect on the profile of lipophilic metabolites. Quantification of metabolites from NMR data provided information for analysis of the effects of treatments on individual metabolites and pathways. This analysis shows some similarities and differences in the effects of the three compounds suggesting possible effects of CA inhibition as well as ER activation on production and utilization of lactate, energy, fatty acids and choline derivatives. Further work will be needed to explore the effects of these types of compounds in hypoxic conditions and on fluxes of metabolites through specific pathways, as well as to further investigate the effects of specific inhibitors of different CA isozymes.

## Supplementary Materials

Supplementary File 1
