# Supplementary material for: Metabolic Effect of Estrogen Receptor Agonists on Breast Cancer Cells in the Presence or Absence of Carbonic Anhydrase Inhibitors
Source: Metabolites. 2016 May 26;6(2):16. doi: 10.3390/metabo6020016 (PMC4931547; doi:10.3390/metabo6020016)
Supplement: Supplementary File 1 [file metabolites-06-00016-s001.zip › metabolites-124279-supplementary/Supplementary Figures.pdf]

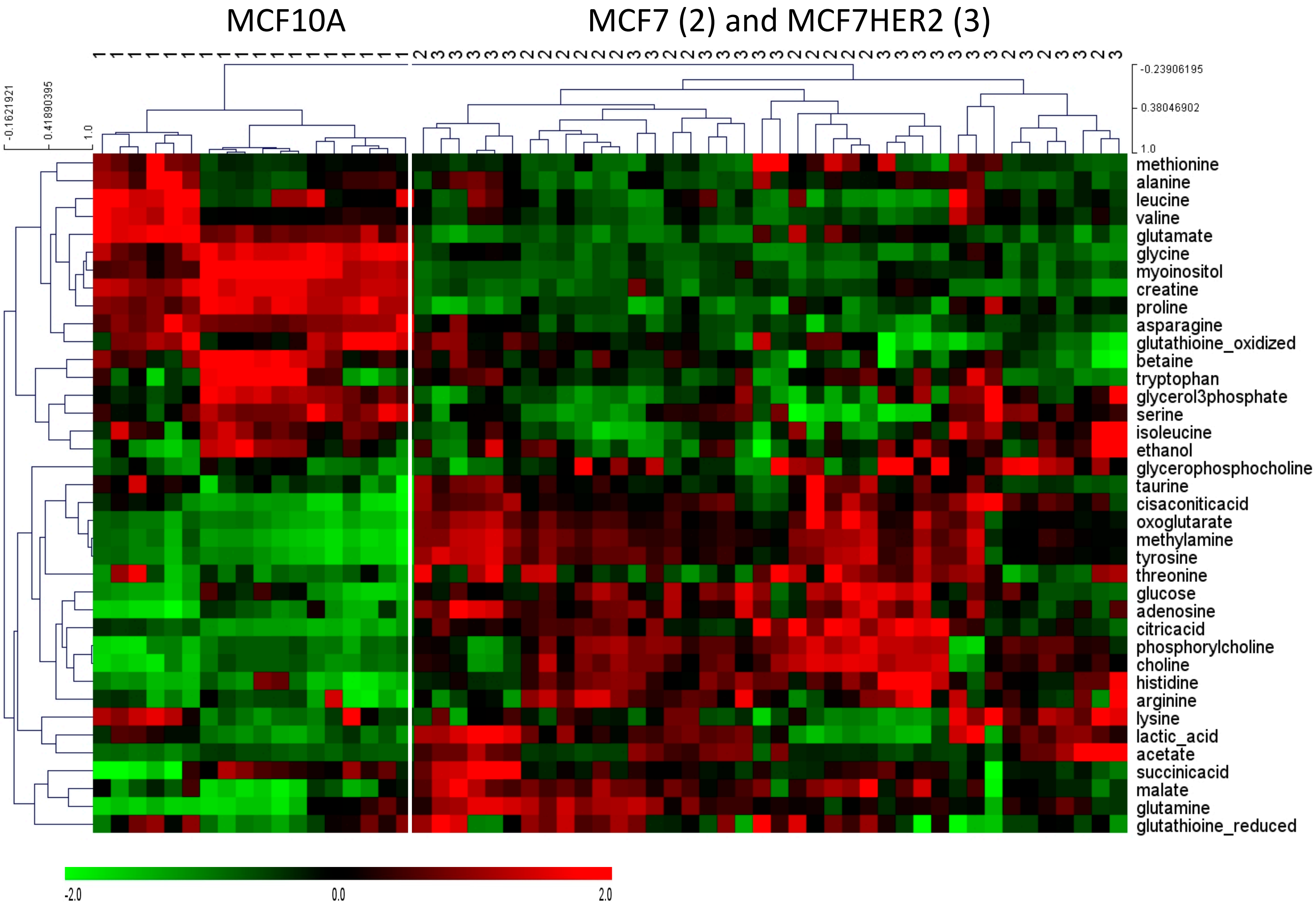

Supplementary Figure S1. Hierarchical cluster analysis of normalized relative metabolite concentrations in all samples

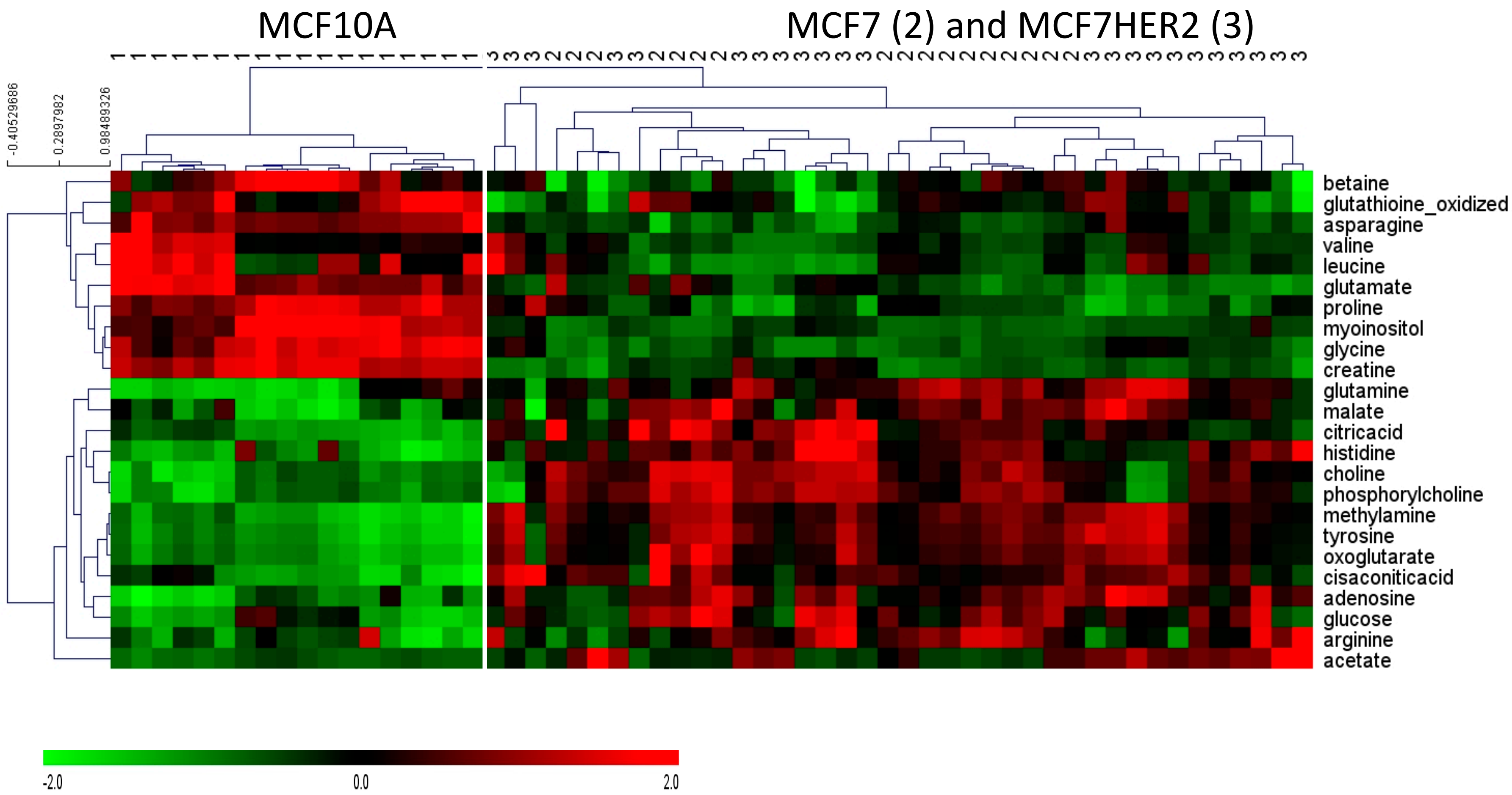

Supplementary Figure S2. Metabolites showing major differences in concentration between all MCF10A and cancer cell lines. Major features were determined using Statistical Analysis for Microarray (SAM) test which performs a feature specific t-tests and computes statistical relevance value that measures strength of the relationship between each feature and a response variable.

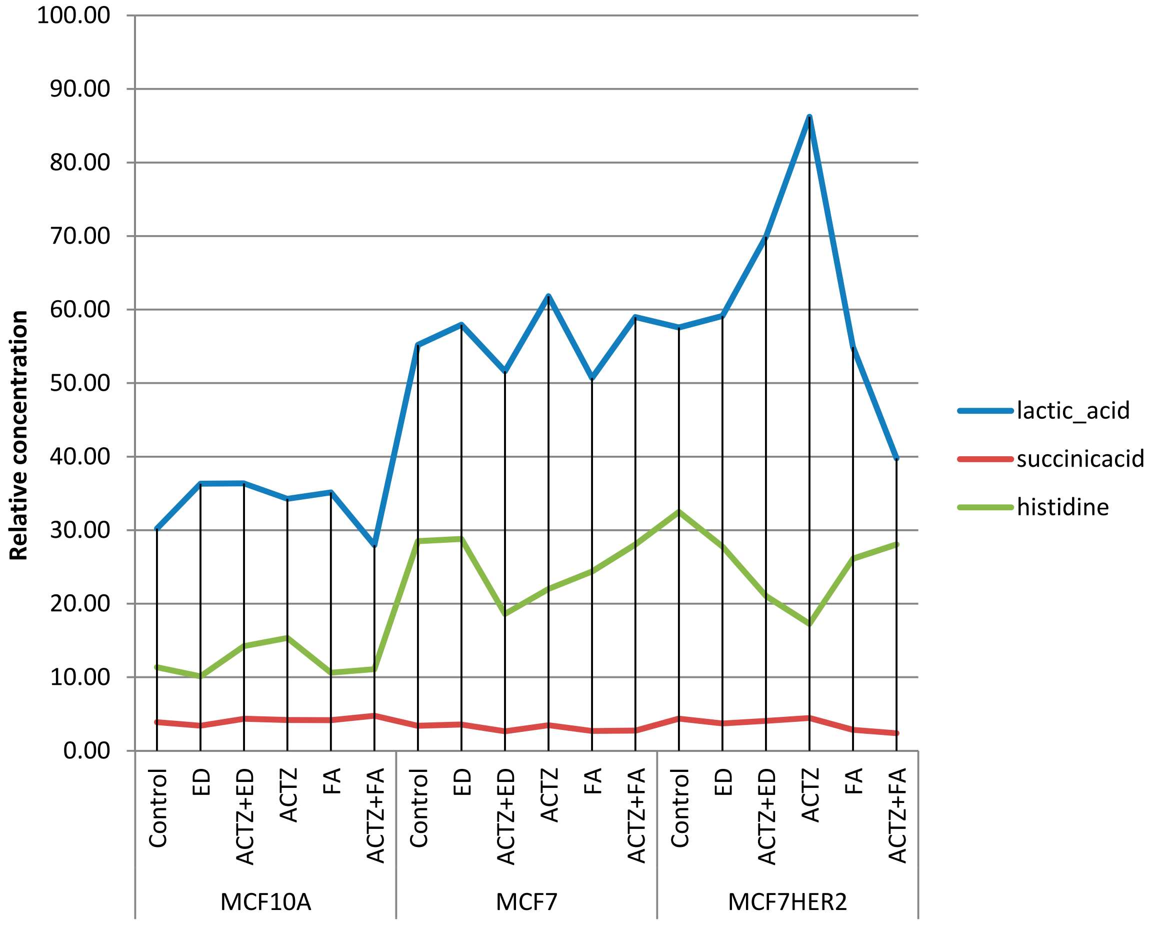

Supplementary Figure S3. Average relative concentration of lactic acid, succinic acid and histidine in different cell lines and treatments.
